# High APRIL Levels Are Associated With Slow Disease Progression and Low Immune Activation in Chronic HIV-1-Infected Patients

**DOI:** 10.3389/fmed.2020.00299

**Published:** 2020-07-17

**Authors:** Yubin Liu, Xiuxia Li, Yang Han, Zhifeng Qiu, Xiaojing Song, Bingxiang Li, Han Zhang, Hongye Wang, Kai Feng, Longding Liu, Jingjing Wang, Ming Sun, Taisheng Li

**Affiliations:** ^1^Department of Infectious Diseases, Peking Union Medical College Hospital, Peking Union Medical College and Chinese Academy of Medical Sciences, Beijing, China; ^2^Institute of Medical Biology, Peking Union Medical College and Chinese Academy of Medical Sciences, Kunming, China; ^3^Yunnan Key Laboratory of Vaccine Research & Development on Severe Infectious Diseases, Kunming, China; ^4^Clinical Immunology Center, Chinese Academy of Medical Sciences, Beijing, China; ^5^School of Medicine, Tsinghua University, Beijing, China

**Keywords:** APRIL, BAFF, HIV-1 disease progression, antibody response, immune activation, functional cells

## Abstract

**Objective:** B-cell-activating factor (BAFF) has been determined to be involved in HIV-1 infection and is correlated with disease progression, while its homologous molecule, a proliferation-inducing ligand (APRIL), is less frequently reported, and its role remains unclear. We aimed to characterize the APRIL levels in subjects with different HIV-1 infection statuses and determine the relationships with disease progression and immune activation.

**Methods:** The plasma levels of APRIL were compared among 17 long-term non-progressors (LTNPs), 17 typical progressors (TPs), 10 ART-treated patients, and 10 healthy donors (HDs). Seventeen LTNPs and a subset of TPs (*n* = 6) who initiated ART were assessed longitudinally. The correlations between the APRIL levels and markers of disease progression, B-cell count and specific antibody response, and markers of immune activation and functional cells were analyzed.

**Results:** The circulating APRIL levels were significantly elevated in the LTNPs relative to the TPs, ART-treated patients, and HDs. The longitudinal investigation revealed that the APRIL levels were decreased during follow-up in the LTNPs. ART did not significantly influence the APRIL levels. The levels of plasma APRIL were negatively correlated with the plasma HIV-1 viral load and cellular HIV-1 DNA levels and positively correlated with the CD4^+^ T-cell count and CD4/CD8 ratio. An inverse correlation was observed between the APRIL and BAFF levels. Furthermore, the APRIL levels were negatively correlated with the frequency of activated CD8^+^ T cells and levels of interferon gamma-induced protein 10 (IP-10) and monocyte chemoattractant protein-1 (MCP-1). Finally, positive correlations were observed among the APRIL levels, the frequency of CD8^+^CD28^+^ T cells, and natural killer (NK) cell count.

**Conclusion:** The APRIL levels were elevated in the LTNPs and negatively correlated with disease progression and immune activation, suggesting likely protective activity in HIV-1 infection.

## Introduction

Persistent immune activation is a key feature of HIV-1 infection in both viremic and antiretroviral therapy (ART)-treated aviremic patients (1). This feature has been linked to suboptimal immune reconstitution and the development of acquired immune deficiency syndrome (AIDS) and non-AIDS events (2–4). In addition to T-cell and myeloid cell activation, abnormal B-cell activation and differentiation are also involved in HIV-1 infection (5–7). B-cell-activating factor (BAFF), which belongs to the TNF family (TNFSF 13b), contributes to B-cell activation and may lead to hypergammaglobulinemia, an impaired humoral response, and the loss of memory B cells (8). High expression levels of BAFF were correlated with the progression of HIV-1 infection (9, 10). A proliferation-inducing ligand (APRIL) also belongs to the TNF family (TNFSF 13a) and shares two receptors with BAFF (11). It has been reported that these molecules have overlapping or similar roles in the pathogenesis of several autoimmune diseases and malignancies (12, 13). However, knowledge regarding the characteristics of APRIL during HIV-1 infection is limited, and the role of APRIL in the pathogenesis of AIDS is less well-defined.

In a longitudinal assessment of HIV-infected individuals with different rates of disease progression, the secretion of APRIL throughout the course of infection in HIV-infected individuals was higher than that in HIV-negative donors. In contrast to BAFF, the circulating APRIL levels were also increased in aviremic and ART-treated rapid progressors (14). However, a cross-sectional comparison among the study groups was not conducted, and the role of APRIL was not well-characterized. When the APRIL levels were measured in primary HIV-infected patients followed for 6 months, no significant difference was observed between healthy donors (HDs) and HIV-positive individuals at any time point analyzed (15). These findings suggest that APRIL may play a different role from BAFF in HIV-1 infection. Therefore, its characteristics deserved further investigation.

In this study, we cross-sectionally quantified APRIL in long-term non-progressors (LTNPs), typical progressors (TPs), ART-treated patients, and HDs. The longitudinal changes in the LTNPs (at enrollment and 4 years later) and a subset of TPs (before and after ART) were also investigated. The relationships between the APRIL levels and markers of disease progression, B-cell count and specific antibody responses, and markers of immune activation and functional cells were analyzed. We sought to characterize the circulating APRIL levels in subjects with different HIV-1 infection statuses and the relationships with disease progression and immune activation.

## Materials and Methods

### Study Subjects

Seventeen HIV-1 LTNPs were selected from the Henan cohort among individuals infected with HIV-1 clade B′ through plasma donation in the 1990s in China. The LTNPs were defined as HIV-1-infected individuals who remained asymptomatic for 10 or more years with CD4^+^ T-cell counts >400 cells/μl in the absence of ART (16, 17). The baseline values were defined as the earliest available values since enrollment (2007), and the latest values were defined as the last values before the time of analysis (2011).

HIV-1 TPs (*n* = 17) matched based on age, sex, infection route, and HIV-1 clade served as controls. The TPs were defined as HIV-1-infected individuals who progressed to AIDS within 7 years post-seroconversion (18). The ART-treated samples (*n* = 10) were collected at a time point with no detectable viral load (<50 copies/ml) for at least 2 years and CD4^+^ T-cell counts >400 cells/μl during ART, and a subset of these patients (*n* = 6) was obtained from on-ART TPs. Blood samples from HDs (*n* = 10) were available for research purposes.

### HIV-1 Viral Load and Measurement

The HIV-1 viral load in plasma samples from infected individuals was determined with a COBAS Ampliprep/TaqMan48 real-time reverse transcriptase polymerase chain reaction (RT-PCR) Test (Roche Diagnostics, Indianapolis, Indiana, USA) according to the manufacturer's instructions. The lower detection limit of the assay was 50 HIV-1 RNA copies/ml.

### HIV-1 DNA Quantification

Cellular HIV-1 DNA in peripheral blood was quantified as previously described (19). In total, 200 μl of whole blood was used to extract cellular DNA (QIAsymphony DNA Mini Kits, Qiagen, Valencia, CA), and HIV DNA was amplified using primers targeting the long terminal repeat gene; concurrently, the housekeeping gene albumin was amplified (real-time HIV Quantitative Detection Kit, Supbio, Guangzhou, China). The total HIV DNA was quantified by using a 7500 Real-time PCR System (Applied Biosystem, USA). All samples were tested in duplicate.

### Cytokine Measurements

We used enzyme-linked immunosorbent assay (ELISA) to measure the plasma levels of APRIL (BioLegend, San Diego, California, USA) and BAFF (R&D Systems, Minneapolis, Minnesota, USA) according to the manufacturer's instructions. The plasma levels of interferon gamma-induced protein-10 (IP-10) and monocyte chemoattractant protein-1 (MCP-1) were measured with a multiplex assay (Human Cytokine/Chemokine Panel I, Millipore, Billerica, Massachusetts, USA) on a Luminex200 platform. Each sample was tested in duplicate, and the results are reported as the mean values.

### Flow Cytometry Analyses

Flow cytometry analyses of peripheral blood lymphocytes were performed as previously described (20). Freshly collected whole blood was incubated with panels of fluorescein isothiocyanate (FITC)/phycoerythrin (PE)/peridinin chlorophyll protein (PerCP)-conjugated antibodies against CD3/CD8/CD4, CD3/CD16CD56/CD19, HLA-DR/CD38/CD8, CD28/CD8/CD4, and isotype controls (Immunotech, Marseilles, France). The lymphocyte phenotype was analyzed by using a three-parameter flow cytometer (Epics XL flow cytometry, Beckman Coulter, USA). Cell counts of lymphocyte subsets were calculated using a dual-platform method with white blood cell (WBC) counts and lymphocyte differentials obtained from routine blood tests of the same specimen.

### Measurement of HIV-1-Specific Antibodies

ELISA was used to measure the HIV-1-specific plasma IgG, IgM, IgA, and subclasses of IgG (IgG1, IgG2, IgG3, and IgG4). HIV-1 antigen (YU2 gp140; Sino Biological, Beijing, China) in phosphate buffered saline (PBS) (pH 7.4) at 0.1 μg/ml was used to coat the plates. The plasma samples were diluted in blocking buffer (1:100 for IgA, IgG2, IgG3, and IgG4; 1:1,000 for IgM; and 1:10,000 for IgG and IgG1). The bound antibodies were detected with horseradish peroxidase (HRP)-conjugated goat anti-human IgG, IgM, and IgA (1:10,000, Abcam) and mouse anti-human IgG1, IgG2 (1:1,000, Abcam), IgG3 (1:1,000, Thermo Fisher, Waltham, Massachusetts, USA), and IgG4 (1:4,000, Abcam). The color reaction was developed using 3,3',5,5'-tetramethylbenzidine (TMB) (Solarbio, Beijing, China) and read at 450 nm using an ELISA microplate reader (Molecular Devices). Each sample was tested in duplicate, and the results are reported as the mean values.

### HIV-1 Neutralization Assay

Neutralizing activity in plasma from HIV-1-infected participants was measured using a luciferase-based assay of TZM-bl cells as previously described (21). The env-pseudotyped viruses used were obtained from a panel of 12 tier 2 and tier 3 clade B (*n* = 4), C (*n* = 4), and CRF_01AE (*n* = 4) viruses (Supplementary Table 1). The HIV-1 envelope pseudoviruses were produced by co-transfecting HEK293T cells with an HIV-1 envelope containing expression vector and an HIV-1 genomic vector (pSG3 delta *env* backbone). Neutralization was detected as a reduction in β-galactosidase reporter gene expression after a single-round infection in TZM-bl cells. The 50% inhibitory dose (ID_50_) was calculated as the plasma dilution causing a 50% reduction in the relative luminescence units (RLU) from the level in the virus control wells after subtracting the RLU in the cell control wells.

### Statistical Analyses

The data are reported as the median (interquartile range, IQR) of the continuous variables and *n* (%) of the categorical variables. Kruskal–Wallis and Dunn's multiple comparison post-tests were used to compare more than two study groups. The paired comparisons were analyzed using a Wilcoxon matched-pairs test. A chi square test was used to compare the categorical data. The correlations were determined by the Spearman rank method. The analyses were performed using Prism 5 software (GraphPad, La Jolla, California, USA). *p* < 0.05 was considered statistically significant.

## Results

### Characteristics of the Participants

**Figure 1:**
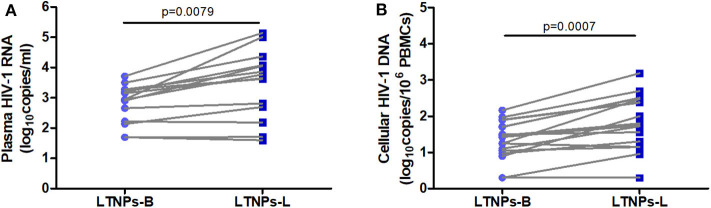
Plasma HIV-1 RNA and cellular HIV-1 DNA viral load in the LTNPs. **(A)** Longitudinal changes in the plasma HIV-1 RNA viral load in the LTNPs. **(B)** Longitudinal changes in the cellular HIV-1 DNA viral load in the LTNPs. LTNPs-B, long-term non-progressors at baseline; LTNPs-L, long-term non-progressors at the latest measurement. Changes over time in the parameters were analyzed using a Wilcoxon signed rank test.

The sociodemographic and clinical characteristics of the study groups are summarized in Table 1. No significant difference was observed among the HIV-1-infected groups in gender, age, mode of HIV-1 acquisition, and HIV-1 subtype. The duration of infection in the LTNPs and ART-treated patients was longer than that in the TPs as expected due to the definition of the patient groups (*p* < 0.001, respectively). The CD4^+^ T-cell count and CD4/CD8 ratio in the LTNPs during follow-up remained stable and were significantly higher compared with those in the TPs (*p* < 0.001, respectively), which increased among those receiving ART. Similarly, the B-cell count in the TPs was lower than that in the LTNPs at baseline (*p* < 0.05) and latest measurement (*p* < 0.001) and was improved in the ART-treated patients. The plasma HIV-1 viral load in the TPs was significantly higher than that in the LTNPs (*p* < 0.001) and decreased to <50 copies/ml among those treated with ART; the LTNPs exhibited elevated plasma viral levels over time (*p* = 0.0079; Figure 1A). In addition, throughout the follow-up duration, the LTNPs experienced an increase in the levels of cellular HIV-1 DNA (*p* = 0.0007; Figure 1B).

**Table 1:** Characteristics of the study participants.

**Characteristic**	**LTNPs (*****n*** **=** **17)**		**TPs (*n* = 17)**	**ART-treated patients (*n* = 10)**	**Healthy adults** **(*n* = 10)**
	**LTNPs-baseline^**a**^**	**LTNPs-latest^**a**^**			
Sex (male)	11 (65%)		7 (41.2%)	6 (60%)	5 (50%)
Age (years)	36 (34–38)	40 (33–56)	34 (30–40)	42 (36–50)	31 (26–37)
HIV risk factor	Plasma donation	Plasma donation	Plasma donation	Plasma donation	NA
HIV-1 subtype	B′	B′	B′	B′	NA
Duration of infection (years)	12 (12–14)	16 (14–21)	7 (6–8)	12.5 (12–16)	NA
CD4^+^ T-cell count (cells/μl)	510 (491–569)	577 (494–640)	36 (26–74)	478 (430–566)	692 (514–1027)
CD4/CD8	0.75 (0.60–0.97)	0.53 (0.46–0.75)	0.11 (0.07–0.26)	0.66 (0.50–0.78)	1.26 (0.92–1.83)
B-cell count (cells/μl)	160 (142–202)	209 (153–238)	114 (67–126)	234 (193–355)	218 (180–313)
Plasma HIV RNA VL (copies/ml)	801(<50–1566)	4179 (51–11,279)	267,358 (57,527–495,615)	<50 (<50– <50)	NA
Cellular HIV-1 DNA VL (copies/10^6^ PBMCs)	26 (11–51)	63 (20–301)	–	–	NA

*Data are expressed as n (%) or median (IQR). LTNP, long-term non-progressors; TPs, typical progressors; ART, antiretroviral therapy; HDs, healthy donors*.

a *Baseline values were defined as the earliest available values since enrollment (2007), and the latest values were defined as the last values (2011) before the time of analysis*.

*NA, not applicable*.

### Cross-Sectional and Longitudinal Analysis of the Plasma APRIL Levels in the Study Participants

**Figure 2:**
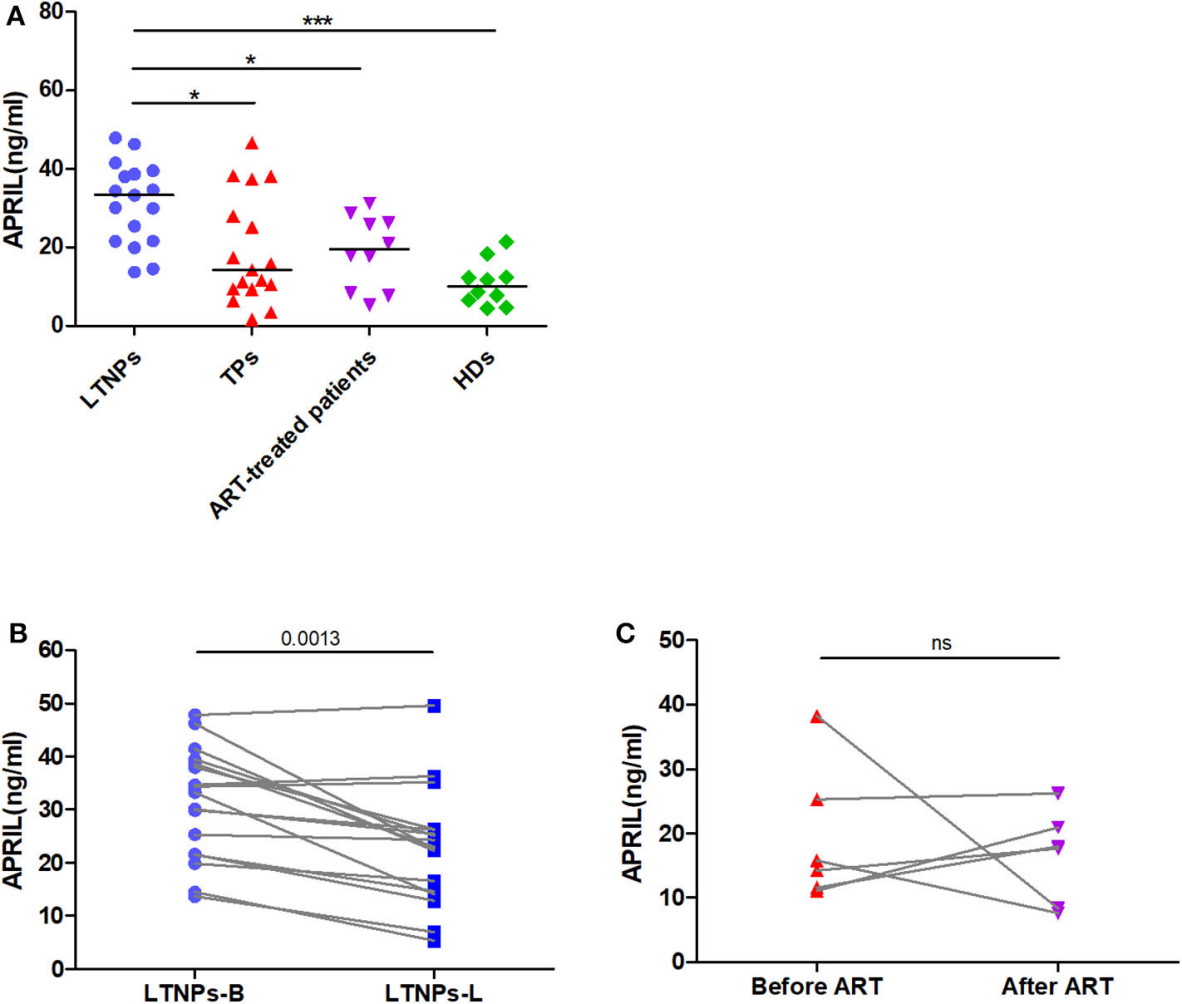
Cross-sectional and longitudinal plasma levels of APRIL in the study participants. **(A)** Plasma levels of APRIL in LTNPs (*n* = 17), TPs (*n* = 17), ART+ (*n* = 10), and HDs (*n* = 10). **(B)** Longitudinal analysis of the APRIL levels over 4 years in the LTNPs (*n* = 17). **(C)** Longitudinal analysis of the APRIL levels in the TPs before and after ART (*n* = 6). LTNPs, long-term non-progressors; TPs, typical progressors; ART, antiretroviral therapy; HDs, healthy donors; LTNPs-B, long-term non-progressors at baseline; LTNPs-L, long-term non-progressors at the latest measurement; ns, not significant. Intergroup comparisons were performed using a Kruskal–Wallis test, followed by Dunn's post-test; paired comparisons were analyzed using a Wilcoxon matched-pairs test (*p < 0.05 and ***p < 0.001).

We first performed a cross-sectional analysis of the circulating APRIL and BAFF levels in the LTNPs, TPs, ART-treated patients, and HIV-1-seronegative individuals. As depicted in Figure 2A, the plasma levels of APRIL in the LTNPs (31.2 ± 10.4 ng/ml) were significantly elevated compared to those in the TPs (19.1 ± 13.9 ng/ml), ART-treated patients (20.9 ± 11.9 ng/ml) and HDs (10.8 ± 5.6 ng/ml). The APRIL levels in the TPs and ART-treated patients were comparable and did not significantly differ from those in the HDs (Figure 2A). The longitudinal analysis of the LTNPs showed that the APRIL levels decreased from 31.2 ± 10.4 ng/ml to 22.7 ± 11.2 ng/ml over the 4-year follow-up (*p* = 0.0013; Figure 2B). In contrast, the changes in the APRIL levels in six TPs receiving ART during follow-up did not show statistical significance (Figure 2C). As expected, the plasma levels of BAFF in the TPs (3,248 ± 1,766 pg/ml) were higher than those in the LTNPs (1,247 ± 250 pg/ml), ART-treated patients (1,462 ± 477 pg/ml), and HDs (1,103 ± 238 pg/ml) (Supplementary Figure 1A). In contrast to APRIL, the BAFF levels in the LTNPs did not significantly change over time, while they significantly decreased in the TPs receiving ART (*p* = 0.0411; Supplementary Figures 1B,C).

### Relationships Between the APRIL Levels and Markers of HIV-1 Disease Progression

**Figure 3:**
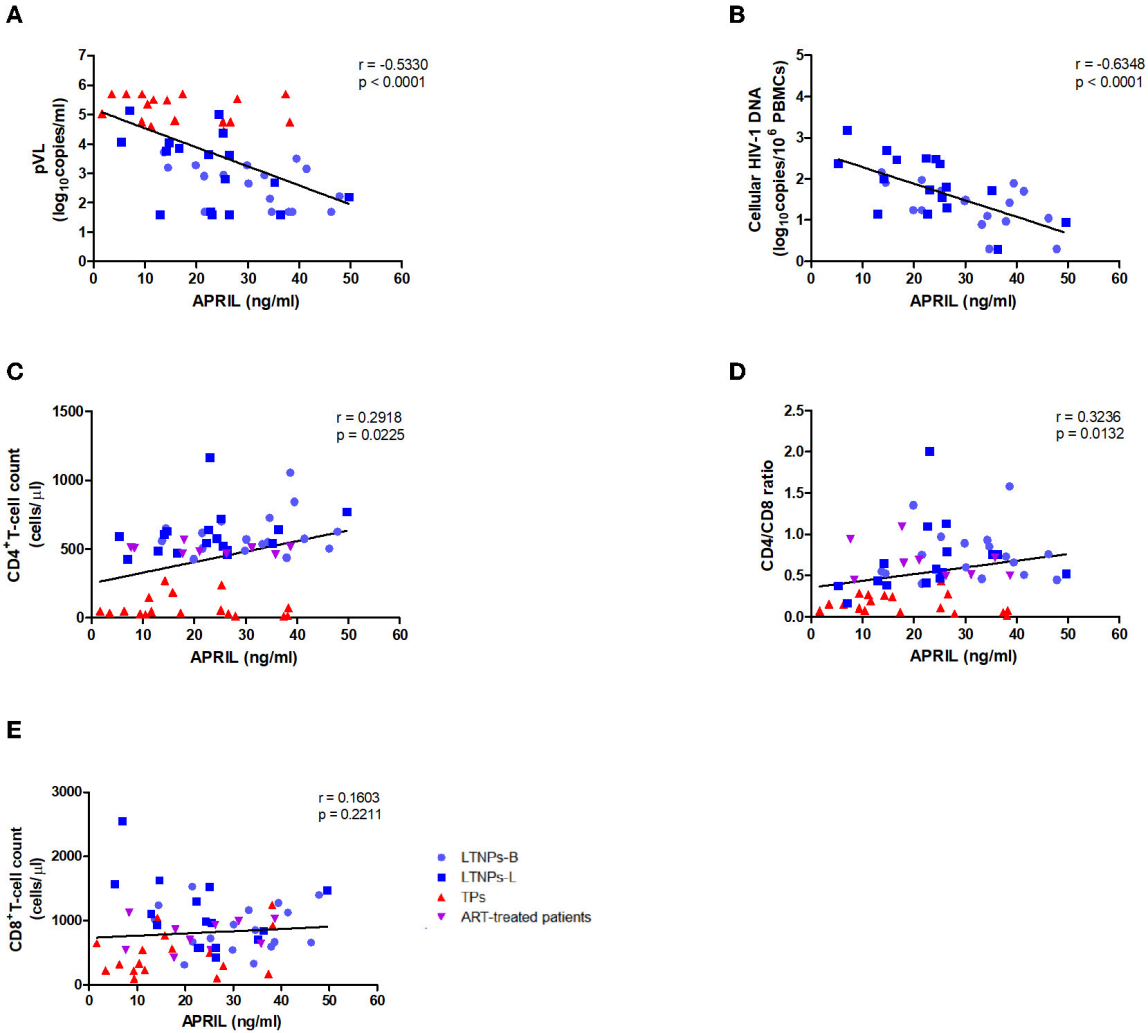
Relationships between the plasma levels of APRIL and markers of HIV-1 disease progression. **(A)** Relationships between the APRIL levels and plasma HIV-1 RNA viral load in untreated HIV-1-infected individuals (*n* = 51). **(B)** Relationships between the APRIL levels and cellular HIV-1 DNA levels in the LTNPs (*n* = 34). **(C)** Relationships between the APRIL levels and CD4^+^ T-cell count in HIV-1-infected individuals (*n* = 61). **(D)** Relationships between the APRIL levels and CD4/CD8 ratio in HIV-1-infected individuals (*n* = 60). **(E)** Relationships between the APRIL levels and CD8^+^ T-cell count in HIV-1-infected individuals (*n* = 60). LTNPs-B, long-term non-progressors at baseline; LTNPs-L, long-term non-progressors at the latest measurement; TPs, typical progressors; ART, antiretroviral therapy. *p* and *r* values were calculated by using Spearman's rank correlation tests.

As higher APRIL levels were observed in the favored clinical status, we first evaluated whether the plasma levels of APRIL might be associated with HIV-1 disease progression. The results illustrated that the APRIL levels were significantly negatively correlated with the plasma HIV-1 RNA viral load in the untreated HIV-1-infected individuals (*r* = −0.5330, *p* < 0.0001; Figure 3A). Similarly, the APRIL levels were significantly negatively correlated with the cellular HIV-1 DNA viral load in the LTNPs (*r* = −0.6348, *p* < 0.0001; Figure 3B). Furthermore, among those with an HIV-1 infection, the APRIL levels were positively associated with both the CD4^+^ T-cell count (*r* = 0.2918, *p* = 0.0225) and CD4/CD8 ratio (*r* = 0.3236; *p* = 0.0132) but not the CD8^+^ T-cell count (Figures 3C–E). In contrast, the BAFF levels were positively correlated with the plasma HIV-1 RNA viral load (*r* = 0.7776, *p* < 0.0001) and cellular HIV-1 DNA viral load (*r* = 0.5027, *p* = 0.0024) and negatively correlated with the CD4^+^ T-cell count (*r* = −0.5749, *p* < 0.0001) and CD4/CD8 ratio (*r* = −0.6877; *p* < 0.0001; Supplementary Figure 2). Thus, the plasma APRIL levels were inversely correlated with HIV-1 disease progression in humans, which differed from BAFF.

### Associations Between the APRIL Levels and HIV-1-Specific Antibody Responses

**Figure 4:**
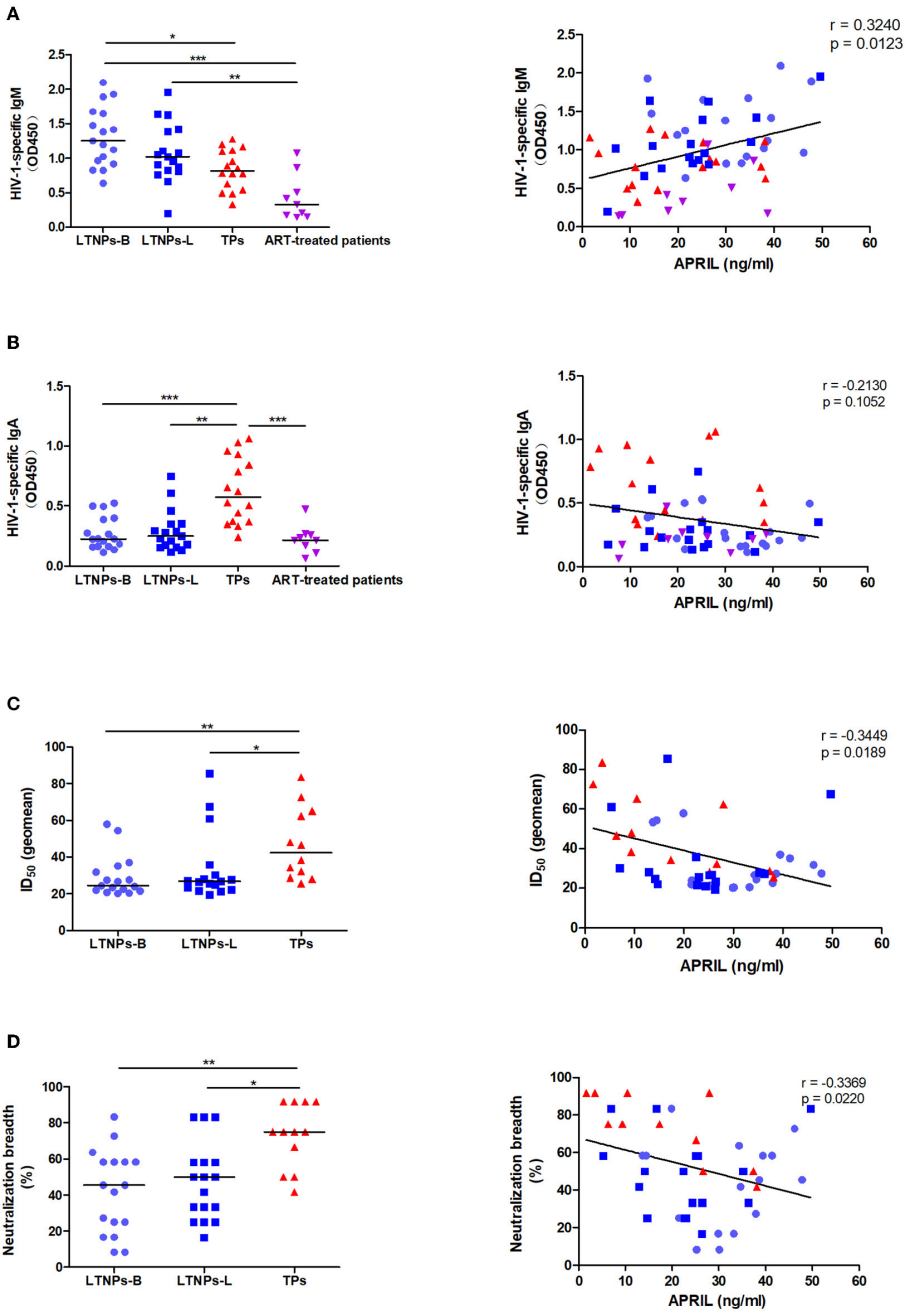
Associations between the plasma levels of APRIL and immunoglobulin production. **(A)** Associations between the APRIL levels and HIV-1-specific IgM in HIV-1-infected individuals (*n* = 59). **(B)** Associations between the APRIL levels and HIV-1-specific IgA in HIV-1-infected individuals (*n* = 59). **(C)** Associations between the APRIL levels and neutralization titers in untreated HIV-1-infected individuals (*n* = 46). **(D)** Associations between the APRIL levels and neutralization breadth in untreated HIV-1-infected individuals (*n* = 46). LTNPs-B, long-term non-progressors at baseline; LTNPs-L, long-term non-progressors at the latest measurement; TPs, typical progressors; ART, antiretroviral therapy; ID_50_, 50% inhibitory dilution. Intergroup comparisons were performed using a Kruskal–Wallis test, followed by Dunn's post-test; *p* and *r* values were calculated by Spearman's rank correlation tests (*p < 0.05, **p < 0.01, and ***p < 0.001).

To investigate the potential immune function of APRIL during HIV-1 infection, we analyzed its relationships with humoral immune responses. We observed that the B-cell count and HIV-1-specific IgG were not associated with the circulating APRIL levels (Supplementary Figures 3A,B). Similarly, no correlation was observed with HIV-1-specific IgG1, IgG2, IgG3, and IgG4 (Supplementary Figures 3C–F). Interestingly, the LTNPs had higher levels of plasma HIV-1-specific IgM, while the plasma HIV-1-specific IgA levels were higher in the TPs, and we found that the APRIL levels were positively correlated with plasma HIV-1-specific IgM (*r* = 0.3240, *p* = 0.0123), especially in the LTNPs at the latest measurement (*r* = 0.5025, *p* = 0.0398), but not plasma HIV-1-specific IgA (Figures 4A,B). We also investigated plasma neutralization activity in the participants, and the results showed that both the neutralization titers and breadth were higher in the TPs and negatively associated with the plasma APRIL levels (*r* = −0.3449, *p* = 0.0189; *r* = −0.3369, *p* = 0.0220, respectively; Figures 4C,D). In contrast, the BAFF levels were negatively correlated with the B-cell count (*r* = −0.4728, *p* = 0.0002) and positively correlated with HIV-1-specific IgG2 (*r* = 0.3782, *p* = 0.0031), IgG4 (*r* = 0.3320, *p* = 0.0109), IgA (*r* = 0.4241, *p* = 0.0008), and the neutralization titers (*r* = 0.4749, *p* = 0.0009) and breadth (*r* = 0.5754, *p* < 0.0001; Supplementary Figure 4). Taken together, compared to BAFF, soluble APRIL played a distinct role in humoral immune responses in HIV-1 infection.

### Correlations Between the APRIL Levels and Markers of Immune Activation

**Figure 5:**
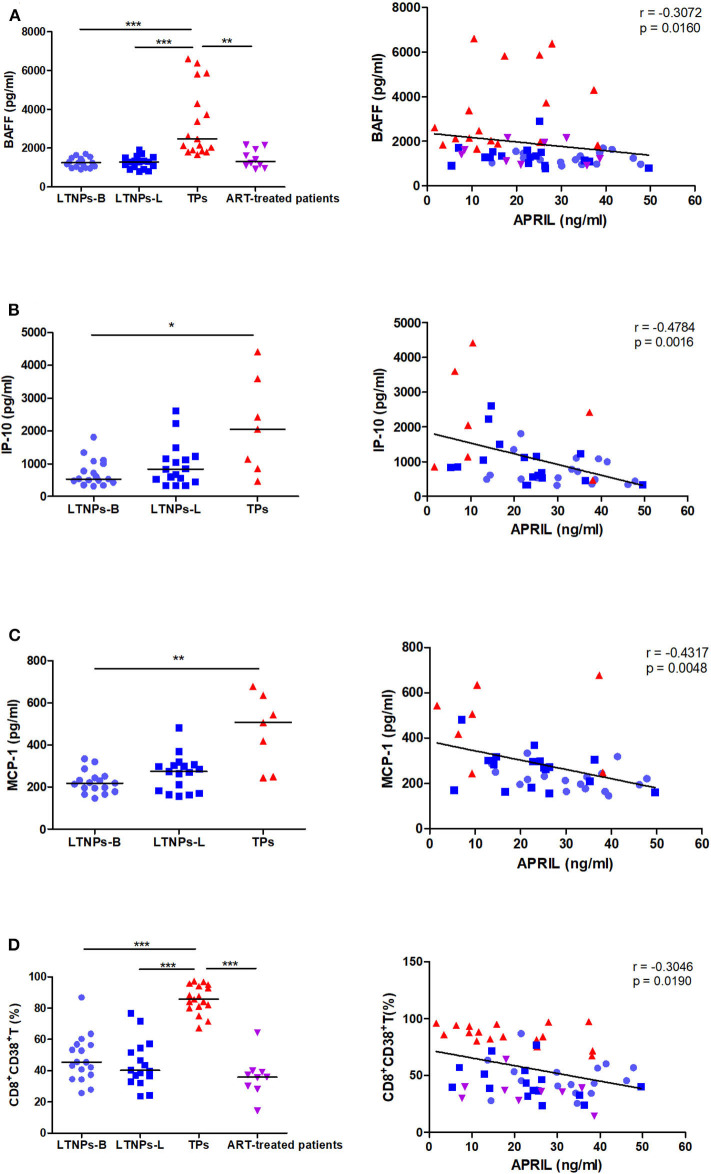
Correlations between the plasma levels of APRIL and markers of immune activation. **(A)** Correlations between the APRIL and BAFF levels in HIV-1-infected individuals (*n* = 61). **(B)** Correlations between the APRIL and IP-10 levels in untreated HIV-1-infected individuals (*n* = 41). **(C)** Correlations between the APRIL and MCP-1 levels in untreated HIV-1-infected individuals (*n* = 41). **(D)** Correlations between APRIL and the proportion of CD8^+^CD38^+^ T cells in HIV-1-infected individuals (*n* = 59). LTNPs-B, long-term non-progressors at baseline; LTNPs-L, long-term non-progressors at the latest measurement; TPs, typical progressors; ART, antiretroviral therapy. Intergroup comparisons were performed using a Kruskal–Wallis test, followed by Dunn's post-test; *p* and *r* values were calculated by Spearman's rank correlation tests (*p < 0.05, **p < 0.01, and ***p < 0.001).

Given the link between HIV-1 disease progression and the humoral immune response, we subsequently assessed the association between the plasma APRIL levels and markers of immune activation. We observed that plasma APRIL was negatively correlated with the BAFF levels in the HIV-1-infected individuals (*r* = −0.3072, *p* = 0.0160; Figure 5A). The APRIL levels were also significantly and negatively correlated with the inflammatory cytokines IP-10 (*r* = −0.4784, *p* = 0.0016) and MCP-1 (*r* = −0.4317, *p* = 0.0048; Figures 5B,C). Additionally, the APRIL levels were negatively correlated with the proportion of activated CD8^+^ T cells, CD8^+^CD38^+^ T cells, in the HIV-1-infected individuals (*r* = −0.3046, *p* = 0.0190; Figure 5D). In the LTNPs-L subgroup, the inverse correlation between the APRIL levels and the percentage of CD8^+^CD38^+^ T cells was more pronounced (*r* = −0.5324, *p* = 0.0338). In contrast, the BAFF levels were positively correlated with IP-10 (*r* = 0.6061, *p* < 0.0001), MCP-1 (*r* = 0.4803, *p* = 0.0015), and the proportion of CD8^+^CD38^+^ T cells (*r* = 0.7241, *p* < 0.0001; Supplementary Figures 5A–C).

### Correlations Between the APRIL Levels and Functional Cells

**Figure 6:**
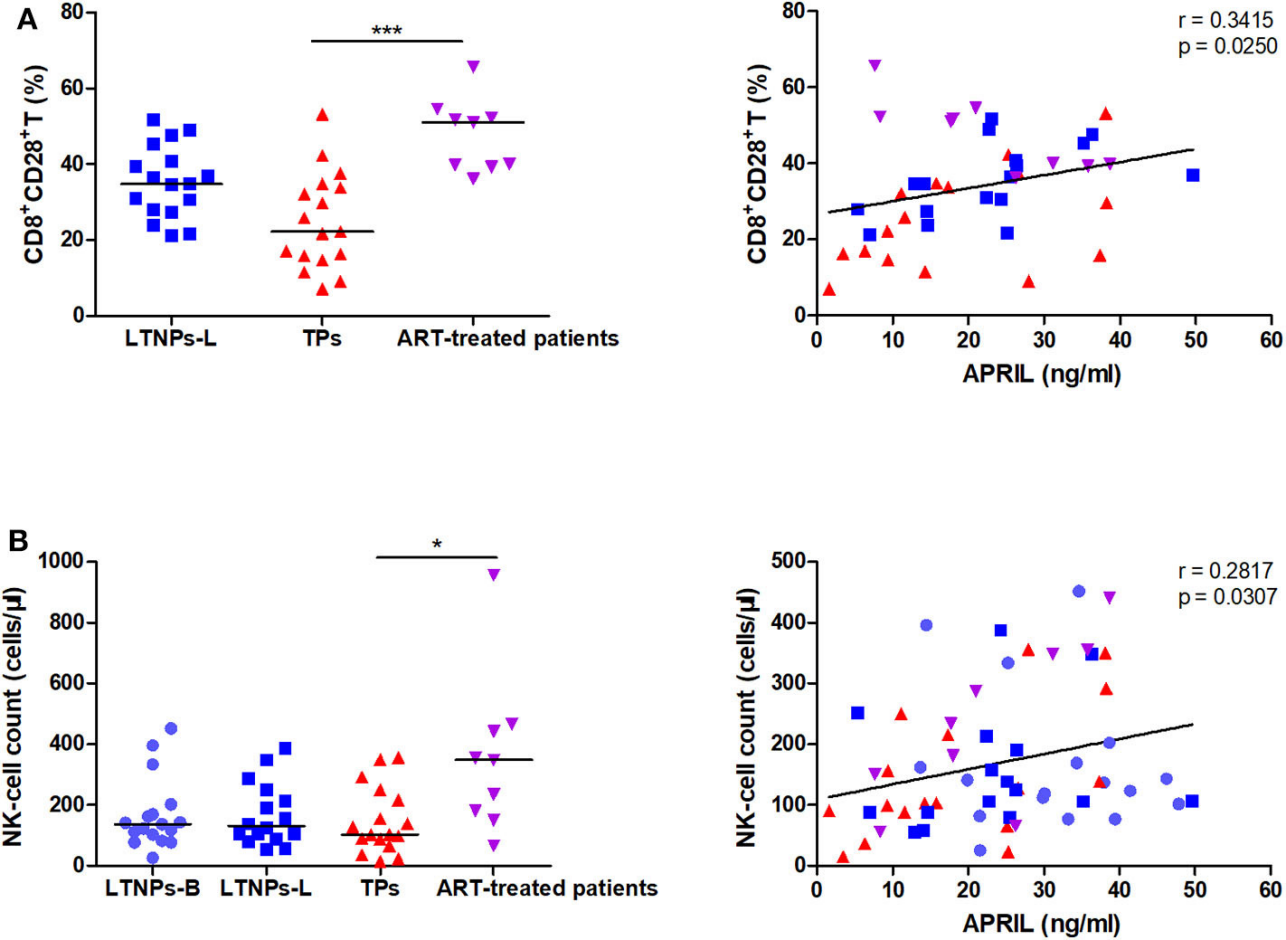
Correlations between the plasma levels of APRIL and functional cells. **(A)** Correlations between APRIL and the proportion of CD8^+^CD28^+^ T cells in HIV-1-infected individuals (*n* = 43). **(B)** Correlations between APRIL and the NK-cell count in HIV-1-infected individuals (*n* = 59). LTNPs-B, long-term non-progressors at baseline; LTNPs-L, long-term non-progressors at the latest measurement; TPs, typical progressors; ART, antiretroviral therapy. Intergroup comparisons were performed using a Kruskal–Wallis test, followed by Dunn's post-test; *p* and *r* values were calculated by Spearman's rank correlation tests (*p < 0.05 and ***p < 0.001).

Furthermore, we found that the circulating APRIL levels were associated with the percentage of functional CD8^+^ T cells, CD8^+^CD28^+^ T cells, in the subgroup of HIV-1-infected individuals (*r* = 0.3415, *p* = 0.0250; Figure 6A). When only the LTNPs were considered, the positive correlation between the APRIL levels and the proportion of CD8^+^CD28^+^ T cells was more significant (*r* = 0.5559, *p* = 0.0254). Moreover, the natural killer (NK) cell count was observed to be correlated with the APRIL levels (*r* = 0.2817, *p* = 0.0307; Figure 6B). This correlation was more significant in the TP group (*r* = 0.5727, *p* = 0.0163). The BAFF levels were negatively correlated with the percentage of CD8^+^CD28^+^ T cells (*r* = −0.5097, *p* = 0.0005), while no correlation was observed with the NK cell count (Supplementary Figures 5D,E). In summary, the soluble APRIL levels were negatively correlated with markers of systemic immune activation and were associated with markers of effector function.

## Discussion

In the present study, we investigated the plasma levels of APRIL in HIV-1-infected subjects with different statuses and observed that the APRIL levels were significantly elevated in the LTNPs. ART did not lead to statistically significant changes in APRIL. In contrast to BAFF, the APRIL levels were negatively associated with markers of HIV-1 disease progression and immune activation and positively correlated with the percentage of functional CD8^+^ T cells and NK cell count, implying that APRIL might mediate a protective response. To the best of our knowledge, this study is the first to focus on APRIL in HIV-1 infection and describe its association with disease progression and immune activation.

Consistent with previous reports, we observed increased circulating BAFF levels in HIV-1-infected subjects, especially in the progressors, and decreased levels in patients receiving ART. We also found associations between the BAFF levels and markers of HIV-1 disease progression and systemic immune activation (9, 15). The BAFF levels were negatively correlated with the B-cell count and this may be attributed, at least in part, to a negative feedback signal from the B cell that was deficient with B-cell depletion during HIV-1 infection (22, 23). In addition, BAFF levels were positively correlated with HIV-1-specific IgG2, IgG4, and IgA, suggesting that as an inflammatory cytokine, BAFF contributes to B-cell activation.

As a homologous molecule of BAFF, APRIL is the 13th member of the tumor necrosis factor superfamily (TNFSF) and is mainly produced by myeloid cells, including dendritic cells, monocytes, macrophages, and neutrophils (24, 25). Specific Toll-like receptor (TLR) ligands, such as CpG and poly I:C, may increase the production of APRIL (14, 26). Mucosal epithelial cells also release APRIL through TLRs and can further increase APRIL production by activating dendritic cells via thymic stromal lymphopoietin (27). In this work, we observed elevated circulating APRIL levels in HIV-1-infected people. Unexpectedly, the LTNPs had the highest levels compared with the TPs, ART-treated patients, and HDs. In previous studies, the data revealed that rapid progressors likely have lower APRIL levels than normal progressors and that the median levels of APRIL in aviremic slow progressors seemed to be higher than those in viremic slow progressors (9). These findings suggest that HIV-1 infection elicits myeloid cells and epithelial cells producing APRIL and that the mechanism might differ from BAFF. We further showed that ART did not significantly influence the circulating APRIL levels. Our results are consistent with those reported by Julie et al., whose study showed that rapid progressors receiving either 3–6 months or 9–12 months of ART had APRIL levels similar to those during the acute (0–3 months) and early (5–8 months) phases of infection (9).

Furthermore, we observed that the APRIL levels were negatively correlated with the plasma HIV-1 RNA viral load and cellular HIV-1 DNA levels and positively correlated with both the CD4^+^ T-cell count and CD4/CD8 ratio. These observations raise questions regarding how APRIL might be involved in slower disease progression.

APRIL has been shown to modulate B-cell homeostasis (28). In our study, no correlation was observed between the plasma APRIL levels and the B-cell count, which is consistent with previous findings showing that APRIL deficiency or overexpression does not lead to significant abnormalities in B-cell development (28, 29). APRIL has been shown to be involved in IgA class switching (30, 31). Surprisingly, no correlation was observed between the soluble APRIL levels and plasma HIV-1-specific IgA. This discrepancy might be explained by the engagement of other factors responsible for isotype switching. It has been shown that TGF-β and IL-21 can induce class switching in human B cells to IgA (32, 33). BAFF and HIV gp120 also affect isotype switching (34, 35), which is elevated in TPs and inversely correlated with APRIL levels. HIV-1 antigens help drive antibody evolution and develop a high level of anti-HIV-1 neutralizing antibodies (36), which explains the negative associations between APRIL and the neutralization titers and breadth. Additionally, we observed a correlation between the soluble APRIL levels and plasma HIV-1-specific IgM, which is supported by previous findings showing that APRIL increases the IgM response in transgenic mice (28). These results suggest that APRIL might be involved in the antiviral response by improving specific IgM levels in HIV-1 infection.

Together with BAFF, APRIL is considered an inflammatory cytokine (15). However, in contrast to BAFF, we showed inverse correlations between the APRIL levels and markers of immune activation and inflammation. Interestingly, a similar trend was observed in some previous studies investigating other disorders. Morel et al. reported that the serum APRIL and BAFF levels were inversely correlated in systemic lupus erythematosus (SLE) (37). Additionally, Charlotte et al. showed that APRIL, but not BAFF, promoted interleukin 10 (IL-10) production and regulatory functions in human B cells to prevent rheumatoid arthritis (RA) and ameliorate established disease. APRIL-stimulated B cells play a regulatory role in T cells to decrease the secretion of TNF-α and IFN-γ from T cells (38). Cynthia et al. further reported that APRIL-induced IL-10-producing regulatory B cells (Bregs) dampened inflammation in experimental autoimmune encephalitis (EAE) and contact hypersensitivity (CHS) models (39). A recent study investigating multiple sclerosis (MS) showed that APRIL mediated an anti-inflammatory response in astrocytes by producing IL-10 to suppress antigen-specific T-cell proliferation and pathogenic cytokine secretion. This protective activity was not shared with BAFF (40). Furthermore, TACI (transmembrane activator and calcium modulator cyclophilin ligand interactor) is required for the regulation of B-cell function as an inhibitory receptor, and TACI is also detected in a subset of activated T cells (41, 42). Thus, it is possible to speculate that APRIL inhibits immune activation by binding TACI and then delivering a negative signal to B cells and T cells in HIV-1 infection. APRIL-induced IL-10 may also play an important role in regulation. Finally, APRIL and BAFF can form heterotrimers, which may be the mechanism through which APRIL reduces BAFF activity (43, 44). The specific mechanism warrants further exploration.

As reported in previous studies, variations in the number of CD8^+^CD28^+^ T cells closely match variations in the number of CD4^+^ T cells in HIV-1 patients (45, 46). We observed a positive correlation between the circulating APRIL levels and the percentage of CD8^+^CD28^+^ T cells, which is consistent with the above finding that APRIL levels are related to the CD4^+^ T-cell count. Moreover, a correlation between circulating APRIL levels and the NK cell count was also observed. In addition to eliminating virus-infected cells through antibody-dependent cellular cytotoxicity (ADCC) (47), NK cells are engaged to induce the optimal maturation of DCs secreting adequate amounts of important regulatory factors, such as IFN-α and interleukin-15 (48). NK cells also actively participate in the control of viral replication by releasing β-chemokines, which can inhibit the entry of HIV-1 into target cells by preventing the binding of CCR5 with a viral envelope (49). Although a positive association with the NK cell count was observed, no direct interplay was demonstrated. More details remain to be determined.

Our work has some limitations. First, due to the scarcity of the LTNP status and restrictions in sample availability, the sample size was small, and certain parameters were tested on a subset of subjects. Additionally, we only detected circulating APRIL during the chronic phase of HIV-1 infection. In the future, we aim to investigate the pattern of APRIL secretion during the acute phase among LTNPs and TPs. Knowledge of the APRIL profile during the early stages of HIV-1 infection might facilitate a better understanding of its role in HIV-1 pathogenesis. Moreover, the total HIV-1-specific IgG, even gag, pol, and vpu-specific IgG, IgM, and IgA, in patients with different disease statuses and their relationships with APRIL need further investigation. Finally, whether and how HIV-1 or virus-induced cell damage directly regulates APRIL expression and how APRIL signaling is involved in viral control and low immune activation are unclear. Further studies are required to determine the mechanism and its significance in immunopathology.

In conclusion, elevated APRIL levels are associated with slow disease progression and low immune activation, revealing its distinct role from BAFF in HIV-1 infection. APRIL is a discriminant biomarker in LTNPs, and its high levels may represent a protective signal in HIV-1 infection. Understanding the implications of elevated APRIL levels might provide new insight into the mechanisms of natural control against HIV-1 infection. The modulation of APRIL expression may be considered for interventional strategies to enhance virus-specific immunity and combat systemic immune activation.

## Data Availability Statement

All datasets generated for this study are included in the article/Supplementary Material.

## Ethics Statement

The studies involving human participants were reviewed and approved by the Institutional Review Board of Peking Union Medical College Hospital (PUMCH). The patients/participants provided their written informed consent to participate in this study.

## Author Contributions

MS, TL, and YL conceived and designed the studies. YL, XL, YH, ZQ, BL, HZ, HW, KF, and JW performed laboratory experiments. YH, ZQ, YL, and XS collected patient samples and collected clinical data. YL, MS, and LL analyzed data. YL, TL, and MS evaluated and interpreted data. YL wrote the manuscript. TL and MS contributed to manuscript revisions. All authors approved the final manuscript.

## Conflict of Interest

The authors declare that the research was conducted in the absence of any commercial or financial relationships that could be construed as a potential conflict of interest.
