# Supplementary material for: High APRIL Levels Are Associated With Slow Disease Progression and Low Immune Activation in Chronic HIV-1-Infected Patients
Source: Front Med (Lausanne). 2020 Jul 17;7:299. doi: 10.3389/fmed.2020.00299 (PMC7396611; doi:10.3389/fmed.2020.00299)
Supplement: Supplementary file 1 [file Data_Sheet_1.pdf]

SUPPLEMENTARY TABLE 1. Viruses used to measure viral neutralizing activity

**Virus**	**Clade**	**Tier**
SC42266.8	B	2
RHPA4259.7	B	2
REJO4541.67	B	2
TRJO4551.58	B	3
Du156.12	C	2
Du172.17	C	2
Du422.1	C	2
ZM55F.PB28a	C	2
2249_13_2_1	CRF_01AE	2
2316_14_22_3	CRF_01AE	2
2149_39_2_2	CRF_01AE	2
2498_40_32_4	CRF_01AE	2

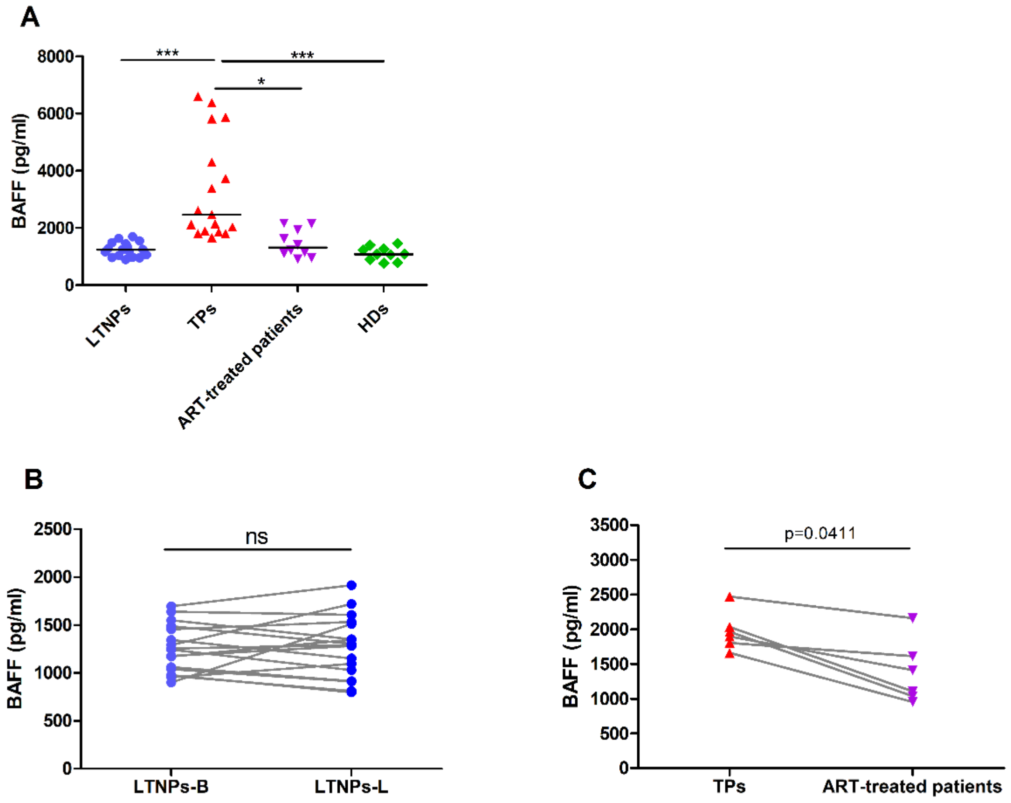

SUPPLEMENTARY FIGURE 1. Cross-sectional and longitudinal plasma levels of BAFF in the study participants. (A) Plasma levels of BAFF in LTNPs (n=17), TPs(n=17), ART+(n=10) and HDs(n=10). (B) Longitudinal analysis of the BAFF levels over 4 years in the LTNPs (n = 17). (C) Longitudinal analysis of the BAFF levels in the TPs before and after ART (n = 6). LTNPs, long-term nonprogressors; TPs, typical progressors; ART, antiretroviral therapy; HDs, healthy donors; LTNPs-B, long-term nonprogressors at baseline; LTNPs-L, long-term nonprogressors at the latest measurement; ns, not significant. Intergroup comparisons were performed using a Kruskal–Wallis test, followed by Dunn's post-test; Paired comparisons were analyzed in a Wilcoxon matched-pairs test. (*$P < 0.05$, ***$P < 0.001$).

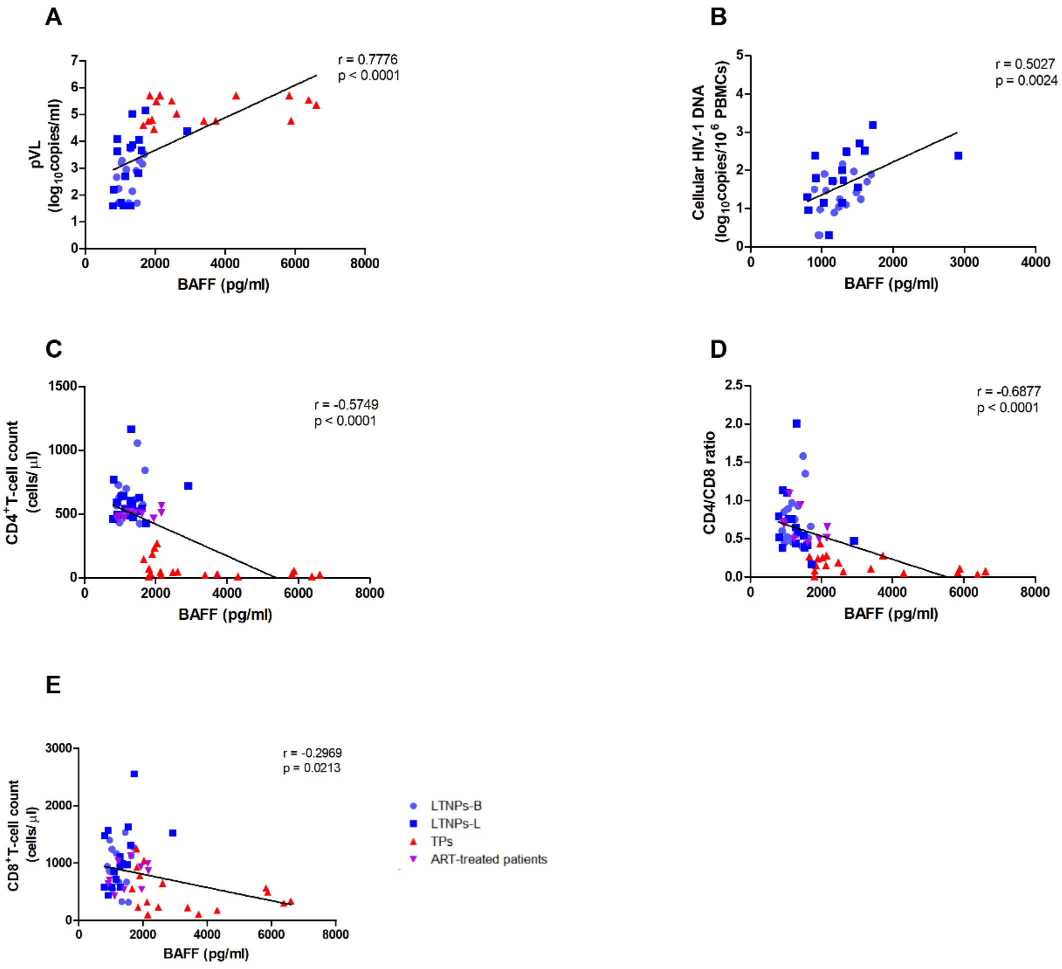

SUPPLEMENTARY FIGURE 2. Relationships between the plasma levels of BAFF and markers of HIV-1 disease progression. (A) Relationships between the BAFF levels and plasma HIV-1 RNA viral load in untreated HIV-1-infected individuals (n=51). (B) Relationships between the BAFF levels and cellular HIV-1 DNA levels in the LTNPs (n=34). (C) Relationships between the BAFF levels and $CD4^{+}$T-cell count in HIV-1-infected individuals (n=61). (D) Relationships between BAFF levels and CD4/CD8 ratio in HIV-1-infected individuals (n=60). (E) Relationships between the BAFF levels and $CD8^{+}$T-cell count in HIV-1-infected individuals (n=60). LTNPs-B, long-term nonprogressors at baseline; LTNPs-L, long-term nonprogressors at the latest measurement; TPs, typical progressors; ART, antiretroviral therapy. P and r values were calculated by Spearman's rank correlation tests.

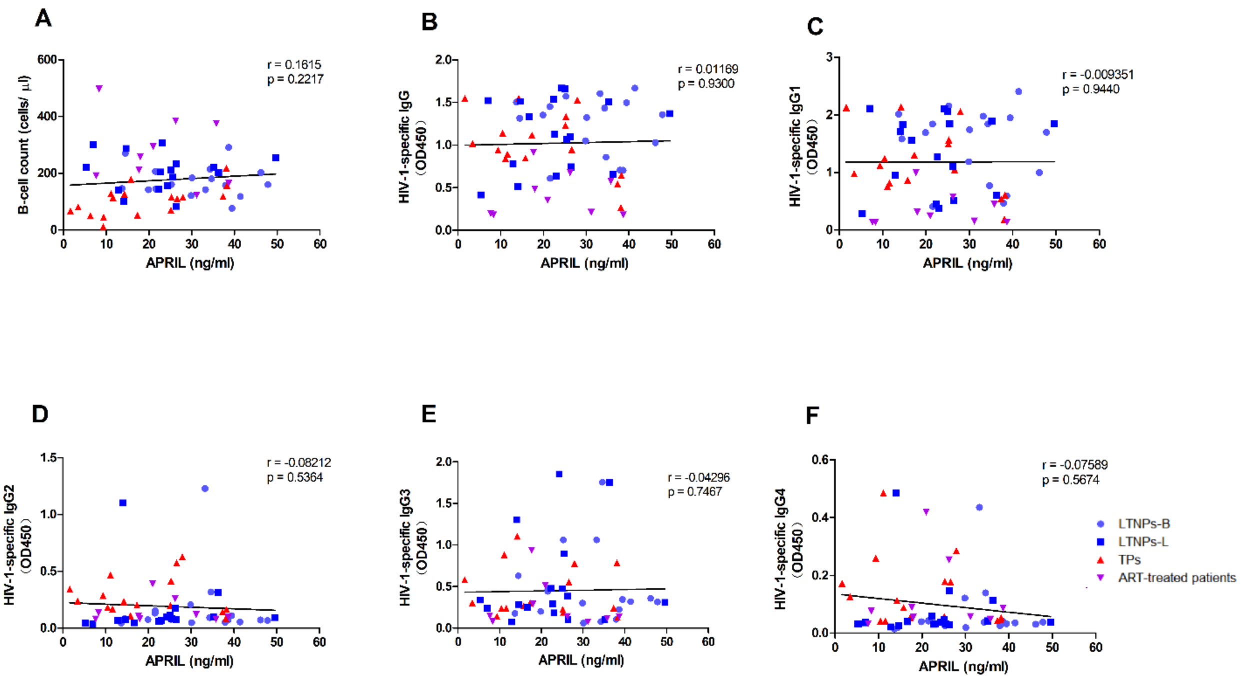

SUPPLEMENTARY FIGURE 3. Associations between the plasma levels of APRIL and humoral immune responses. Associations between the APRIL levels and B-cell count (A), IgG (B), IgG1 (C), IgG2 (D), IgG3 (E) and IgG4 (F) in HIV-1-infected individuals (n=59). LTNPs-B, long-term nonprogressors at baseline; LTNPs-L, long-term nonprogressors at the latest measurement; TPs, typical progressors; ART, antiretroviral therapy. P and r values were calculated by Spearman's rank correlation tests.

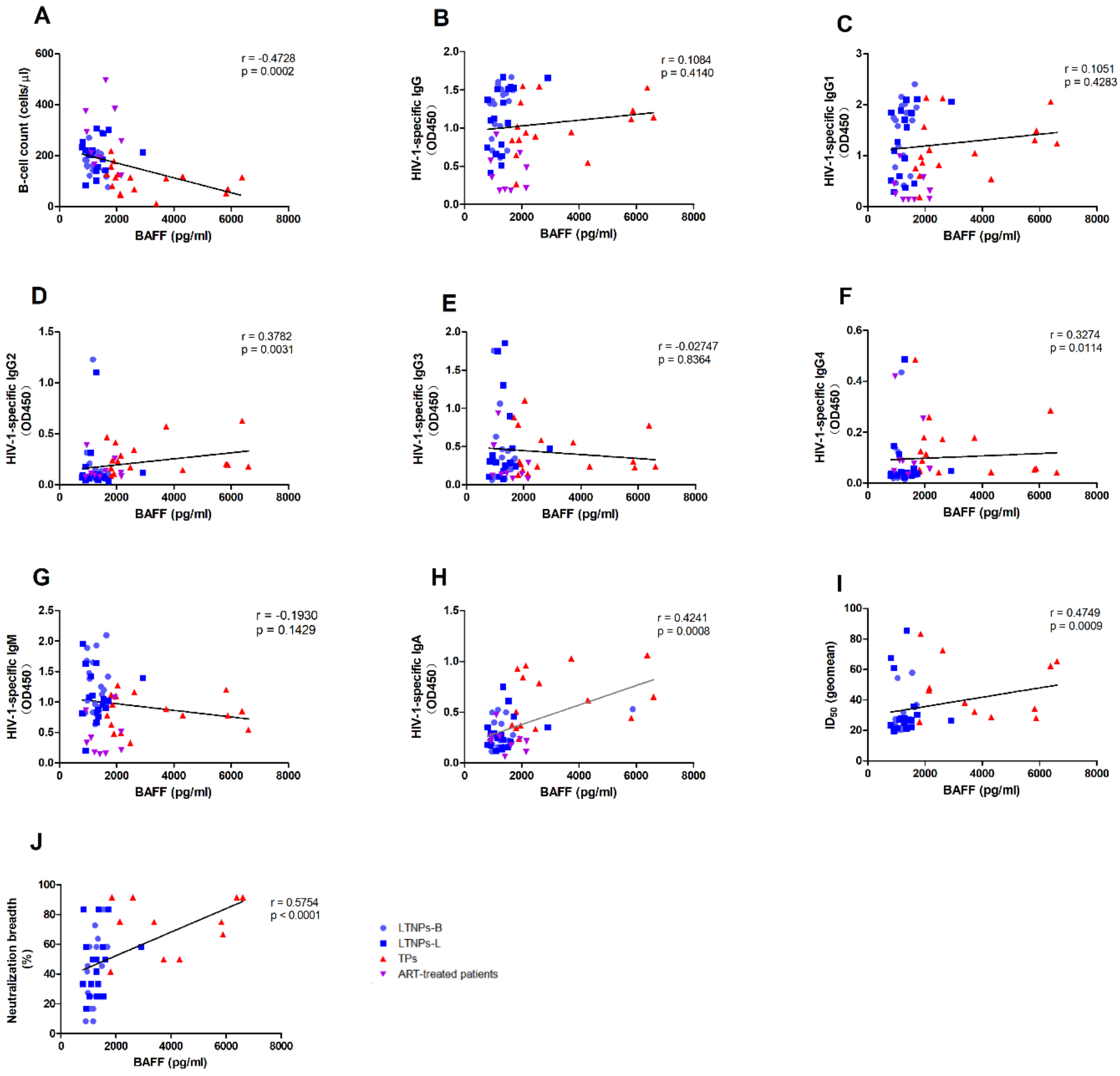

SUPPLEMENTARY FIGURE 4. Associations between the plasma levels of BAFF and B cell and immunoglobulin production. (A) Associations between the BAFF levels and B-cell count in HIV-1-infected individuals (n=59). (B-H) Associations between the BAFF levels and HIV-1-specific IgG, IgG1, IgG2, IgG3, IgG4, IgM and IgA in HIV-1-infected individuals (n=59). (I-J) Associations between the BAFF levels and neutralization titers and breadth in untreated HIV-1-infected individuals (n=46). LTNPs-B, long-term nonprogressors at baseline; LTNPs-L, long-term nonprogressors at the latest measurement; TPs, typical progressors; ART, antiretroviral therapy; ID50, 50% inhibitory dilution. P and r values were calculated by Spearman's rank correlation tests.

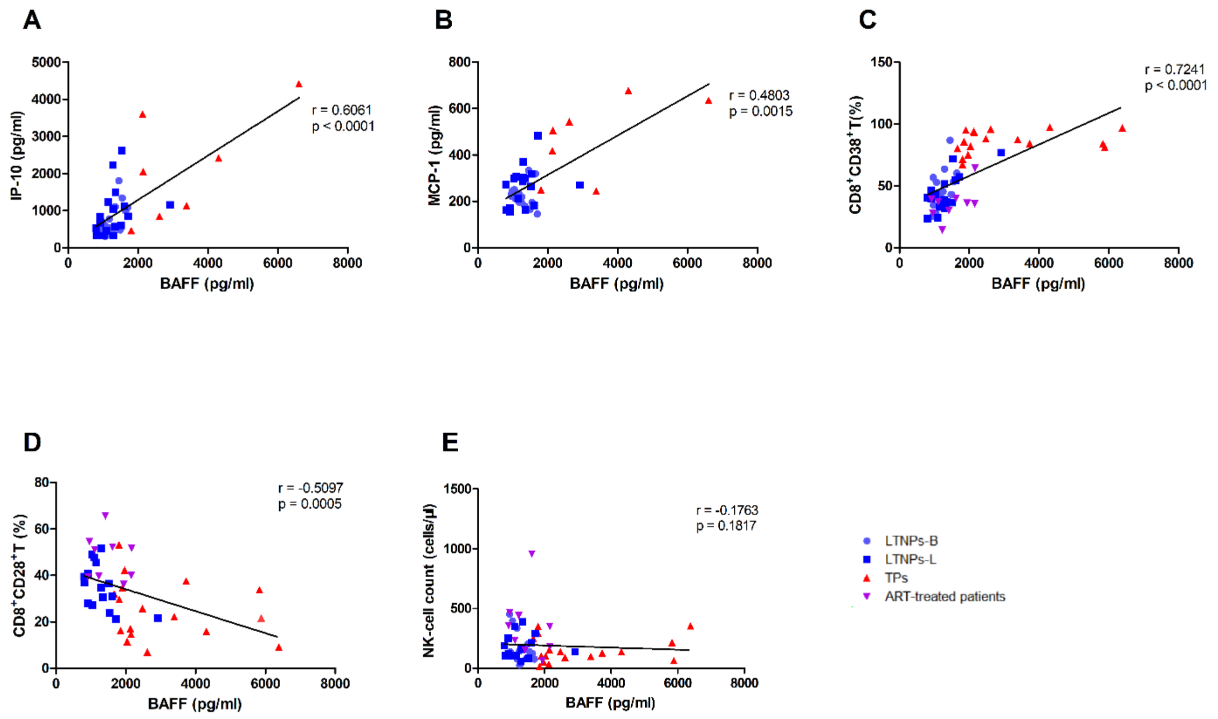

SUPPLEMENTARY FIGURE 5. Correlations the between plasma levels of BAFF and markers of immune activation and functional cells. (A) Correlations between the BAFF and IP-10 levels in untreated HIV-1-infected individuals (n=41). (B) Correlations between the BAFF and MCP-1 levels in untreated HIV-1-infected individuals (n=41). (C) Correlations between BAFF and the proportion of $CD8^{+}CD38^{+}T$ cells in HIV-1-infected individuals (n=59). (D) Correlations between BAFF and the proportion of $CD8^{+}CD28^{+}T$ cells in HIV-1-infected individuals (n=43). (E) Correlations between BAFF and the NK-cell count in HIV-1-infected individuals (n=59). LTNPs-B, long-term nonprogressors at baseline; LTNPs-L, long-term nonprogressors at the latest measurement; TPs, typical progressors; ART, antiretroviral therapy. P and r values were calculated by Spearman's rank correlation tests.
